# Virulence effector SidJ evolution in *Legionella pneumophila* is driven by positive selection and intragenic recombination

**DOI:** 10.7717/peerj.12000

**Published:** 2021-08-17

**Authors:** Xiao-Yong Zhan, Jin-Lei Yang, Xuefu Zhou, Yi-Chao Qian, Ke Huang, Honghua Sun, Huacheng Wang, Yang Leng, Bihui Huang, Yulong He

**Affiliations:** The Seventh Affiliated Hospital, Sun Yat-sen University, Shenzhen, China

**Keywords:** *Legionella pneumophila*, Virulence effector, SidJ, Evolution, Adaptive evolution, Intragenic recombination, Positive selection

## Abstract

Effector proteins translocated by the Dot/Icm type IV secretion system determine the virulence of *Legionella pneumophila* (*L. pneumophila*). Among these effectors, members of the SidE family (SidEs) regulate several cellular processes through a unique phosphoribosyl ubiquitination mechanism mediated by another effector, SidJ. Host-cell calmodulin (CaM) activates SidJ to glutamylate the SidEs of ubiquitin (Ub) ligases and to make a balanced Ub ligase activity. Given the central role of SidJ in this regulatory process, studying the nature of evolution of *sidJ* is important to understand the virulence of *L. pneumophila* and the interaction between the bacteria and its hosts. By studying *sidJ* from a large number of *L. pneumophila* strains and using various molecular evolution algorithms, we demonstrated that intragenic recombination drove the evolution of *sidJ* and contributed to *sidJ* diversification. Additionally, we showed that four codons of *sidJ* which are located in the N-terminal (NTD) (codons 58 and 200) and C-terminal (CTD) (codons 868 and 869) domains, but not in the kinase domain (KD) had been subjected to strong positive selection pressure, and variable mutation profiles of these codons were identified. Protein structural modeling of SidJ provided possible explanations for these mutations. Codons 868 and 869 mutations might engage in regulating the interactions of SidJ with CaM through hydrogen bonds and affect the CaM docking to SidJ. Mutation in codon 58 of SidJ might affect the distribution of main-chain atoms that are associated with the interaction with CaM. In contrast, mutations in codon 200 might influence the *α*-helix stability in the NTD. These mutations might be important to balance Ub ligase activity for different *L. pneumophila* hosts. This study first reported that intragenic recombination and positive Darwinian selection both shaped the genetic plasticity of *sidJ*, contributing to a deeper understanding of the adaptive mechanisms of this intracellular bacterium to different hosts.

## Introduction

*Legionella pneumophila* (*L. pneumophila*) is the most common causative agent of legionellosis, which manifests as atypical pneumonia or non-pneumonia type illnesses, *e.g.*, Pontiac fever (Fields, Benson & Besser, 2002). As a pathogenic Gram-negative bacterium, *L. pneumophila* is widely present in natural environments including natural water and soil sources, in which free-living amoebae is its natural host (Albert-Weissenberger, Cazalet & Buchrieser, 2007; Van Heijnsbergen et al., 2014). From natural environments, *L. pneumophila* can colonize artificial environments (*e.g.*, cooling towers and hot-water systems), and then spread in aerosols, infecting the susceptible person. As an intracellular parasite for protozoa, *L. pneumophila* infects mammalian host cells using similar mechanisms: phagocytosis or macropinocytosis (De Carvalho, Barrias & De Souza, 2015; Horwitz, 1984; Peracino, Balest & Bozzaro, 2010). After entering the host cells, *L. pneumophila* creates an intracellular niche named Legionella-containing vacuoles (LCVs), which are resistant to the acidification and LCV-lysosome fusion, and permissive for its replication (Isberg, OConnor & Heidtman, 2009). In this process, host-cell functions are modulated by hundreds of effector proteins encoded by the *L. pneumophila* genome and delivered by *L. pneumophila* type IV Dot/Icm secretion system (Hubber & Roy, 2010; Segal, Feldman & Zusman, 2005). The genomes of *L. pneumophila* consist of a single circular chromosome of about 3.4 Mb (Gomez-Valero et al., 2011). Some *L. pneumophila* strains (*e.g.*, *Paris*, *Lens* and *Lorraine*) also contain a plasmid (Gomez-Valero et al., 2011). The number of genes in *L. pneumophila* chromosome is about 3,000, of which, 98%–99% are protein-coding genes and about 300 are the type IV Dot/Icm effectors (Gomez-Valero et al., 2011). As a facultatively pathogenic bacterium interacting with free-living amoebae, *L. pneumophila* exhibit a genome larger than their close relatives such as *Coxiella burnetii* and *Francisella tularensis* due to gene conservation and acquisition (Moliner, Fournier & Raoult, 2010). This was also proved by Gomez-Valero et al. (2019) that the number of gene gain events in 2,837 representative proteins of genus *Legionella* considerably exceeded the number of loss events.

Functional redundancy among groups of effector proteins is required for *L. pneumophila* to survive in different host cells (Newton et al., 2010; Richards et al., 2013; Shames et al., 2017). However, only a few of these proteins are necessary for intracellular replication, and elimination of numerous effector genes rarely leads to detectable defects in intracellular growth (O’Connor et al., 2011). There were some critical components for both intracellular growth and disease within animals that have been identified in *L. pneumophila*, including SdhA, SidJ, and AnkB (Al-Khodor et al., 2008; Anand, Choi & Isberg, 2020; Harding et al., 2013; Jeong, Sexton & Vogel, 2015; Liu & Luo, 2007). SidJ, encoded by a 2,622 to 2,628 bp length gene, is a member of the Dot/Icm effector and plays a key role in regulating several host cellular processes and pathways through another effector member named SidE family (SidEs), including SdeA (1,499 aa), SdeB (1,920 aa), SdeC (1,533 aa) and SidE (1,495 aa) (Liu & Luo, 2007). These proteins are encoded by genes with lengths of 4,497 bp, 5,760 bp, 4,599 bp, and 4,485 bp respectively. SidEs localize to the cytoplasmic face of the LCV in the early stages of *L. pneumophila* infection. They are required for the mono-ADP-ribosyltransferase activity involved in ubiquitin activation, which is regulated by SidJ glutamylase activity. Such activity is subsequently modulated by the eukaryote-specific protein calmodulin (CaM) *via* binding (Gan et al., 2019; Liu & Luo, 2007). SidJ catalyzes glutamylation of SidEs (Bhogaraju et al., 2019; Black et al., 2019), and in turn, inhibits their unrestrained ubiquitin (Ub) ligase activity; which is shown to be harmful to the host by poisoning the cellular Ub pool and possibly blocking the action of other *L. pneumophila* effectors that manipulate the host Ub machinery (Bhogaraju et al., 2016). Three functional domains of SidJ were identified in a previous report, including a kinase domain (KD) in the center of protein spanning residues 336 to 593, which forms a catalytic structure and an N-terminal (NTD) and C-terminal domains (CTD) (Black et al., 2019). Given the crucial role of SidJ in this regulation network, studying the nature of evolution of *sidJ* is of great importance to understand the virulence in *L. pneumophila* and the interaction between the bacteria and its hosts. A study on 32 unrelated strains of *L. pneumophila* revealed that recombination was an important strategy in the evolutionary adaptive process and played an active role in *sidJ* genetic plasticity (Costa et al., 2014). Recombination of *sidJ* might also provide a broad-host-range for *L. pneumophila* by preventing host specialization and contributing to the resilience of the species (Costa et al., 2014). Besides recombination, selection was another fundamental evolutionary force that shaped DNA sequence variation. Interaction between recombination and natural selection within a gene can either increase or decrease sequence diversity. Moreover, recombination can generate genetic variation, which is tested by natural selection, and as such, it plays an important role in fueling adaptive evolution (Jouet, McMullan & Van Oosterhout, 2015).

The ultimate goal of this work is to understand the underlying patterns in the evolution of the *sidJ* gene of *L. pneumophila* during its lifecycle (*e.g.*, infect the amoebae *via* amoebal attack and present a sympatric lifestyle) through identifying individual codons under positive selection. We utilized various algorithms to identify the molecular evolution patterns of *sidJ* in a relatively large number of *L. pneumophila* strains. It is shown here both intragenic recombination and positive selection drove the adaptive evolution of *sidJ* and shaped its genetic plasticity. Codons of *sidJ* that were identified to experience positive selection might play key roles in regulating the binding affinity of SidJ to CaM; and thus, change the glutamylases activation of SidJ, which might, in turn, manipulate the host Ub machinery balance. This reticular regulation network might be an important strategy for the survival and adaptability of *L. pneumophila* to variable host cells.

## Materials and Methods

### *L.pneumophila* strains

One hundred and sixteen *L. penumophila* strains were enrolled in this study. These strains were isolated from 1947 to 2016, from different environmental and clinical sources. Full-length sequences of *sidJ* were captured from the whole genome of the strains. The detailed information of these strains including the sources and place of isolation, the collection dates, the NCBI biosample, and the sequence accession numbers were summarized in Table S1. Some of these strains are defined as subspecies (*subsp. pneumophila*, *subsp. fraseri*, *subsp. pascullei,* and *subsp. raphaeli*) of *L. pneumophila,* and belong to different serogroups (sgs) including sg1, sg4, sg5, sg8, sg11, etc. based on literature report (Kozak-Muiznieks et al., 2018).

### Sequence and phylogenetic analysis

The *sidJ* gene sequences from the 116 *L. pneumophila* strains were manually checked for integrity and were aligned by MEGA X software using Muscle (codons) algorithms (Kumar et al., 2018). Allele type and DNA sequence polymorphism analyses were performed by DnaSP 6.12.03 (Rozas et al., 2017). The most appropriate model for *sidJ* nucleotide or SidJ amino acid substitution was determined by the model finder module of MEGA X and using the Akaike Information Criterion (AIC) (Posada & Buckley, 2004). An unrooted phylogenetic tree of the *sidJ* alleles was constructed using MEGA X, inferring the evolutionary history using the Maximum Likelihood (ML) method and Hasegawa-Kishino-Yano model with gamma distribution (HKY+G) (Hasegawa, Kishino & Yano, 1985). Initial trees were obtained automatically by applying the Neighbor-Joining and BioNJ algorithms to a matrix of pairwise distances estimated using the Maximum Composite Likelihood (MCL) approach. The evolutionary history of SidJ protein was inferred by using the Maximum Likelihood method and the JTT matrix-based model (Jones, Taylor & Thornton, 1992). Initial tree(s) for the heuristic search were obtained automatically by applying Neighbor-Join and BioNJ algorithms to a matrix of pairwise distances estimated using the JTT model, and then selecting the topology with a superior log-likelihood value. A discrete Gamma distribution was used to model evolutionary rate differences among sites. Bootstrap values were estimated using 1000 replications.

### Molecular evolution analysis

The neighbor-net analysis was performed and split networks were constructed with algorithms implemented in SplitsTree4 software (version 4.14.4) (Huson & Bryant, 2006). A reticulate network tree was prepared to show the relationships among *sidJ* alleles and to visualize possible recombination events. Pairwise homoplasy index (Phi) tests were used to calculate a measure of statistical significance for recombination and a cutoff value was set at 0.05 (Bruen, Philippe & Bryant, 2006). The *sidJ* allele sequences were screened by RDP4 to detect intragenic recombination (Martin et al., 2015). Six methods (RDP Martin & Rybicki, 2000), GENECONV, BootScan (Martin et al., 2005), MaxChi (Smith, 1992), Chimaera (Posada, 2002), and SiScan (Gibbs, Armstrong & Gibbs, 2000) implemented in the RDP4 were utilized. Potential recombination events (PREs) were defined as those identified by at least four methods. Common settings for all the methods were as following: sequences were considered as linear, statistical significance was set at *P* < 0.05, Bonferroni correction was used to correct *P*-values for multiple comparisons, phylogenetic evidence was required, and breakpoints were polished. Genetic diversity of the *sidJ* alleles was investigated by using DnaSP 6.12.03 (Rozas et al., 2017).

### Population genetics analysis

DnaSP 6.12.03 was used to perform genetic diversity analyses for the *sidJ* alleles (Librado & Rozas, 2009; Rozas et al., 2009). Tajima’s D, Fu, and Li’s D* and F* tests were employed to verify the neutrality hypothesis of *sidJ* as previously described by our research group (Zhan & Zhu, 2017). These analyses were carried out using DnaSP 6.12.03 (Rozas et al., 2017). A statistical significance cutoff was set at 0.05 for all the tests. Nonsynonymous and synonymous mutations of *sidJ* were calculated using the MEGA X software package (Kumar et al., 2018). A parsimony network of *sidJ* alleles was created using PopART software (http://popart.otago.ac.nz) (Clement, Posada & Crandall, 2000). The demographic history of *sidJ* was inferred by analyzing the mismatch distribution of pairwise nucleotide differences in the *sidJ* alleles, which was carried out by an algorithm implemented in Arlequin3.5 (Excoffier & Lischer, 2010). Expected values for a model of constant *sidJ* allele population size were calculated and plotted against the observed values. Harpending’s raggedness index and the sum of squared deviations (SSD), as implemented in Arlequin3.5, were used to evaluate Rogers’ sudden expansion model, which fits a unimodal mismatch distribution (Rogers & Harpending, 1992).

### Analysis of positive selection at the codon level

The positive selection pressure operating on the *L. pneumophila sidJ* gene was investigated using the Maximum Likelihood (ML) method by a visual tool of codeml software program (Bielawski, Baker & Mingrone, 2016), EasyCodeML (Gao et al., 2019). First, the topologies of the ML trees of *sidJ* alleles were generated by MEGA X as mentioned above, for the subsequent selection analysis. Then, three nested models (M3 *vs.* M0, M2a *vs.* M1a, and M8 *vs.* M7) were compared, and the likelihood ratio tests (LRTs) were applied to assess the best fit of codons. Model fitting was performed using multiple seed values for *dN/dS*. SidJ codon frequencies were assumed using the F3x4 model. When the LRT was significant (*P* < 0.05), Bayes empirical Bayes (BEB) (M8 model) and Naive Empirical Bayes (NEB) methods (M3 and M2a models) were used to identify codons that evolved under positive selection based on a posterior probability of more than 0.95. Positive selection was inferred when the individual site or codon had a ratio of nonsynonymous to synonymous mutations greater than one (*ω* > 1). To omit the influence of intragenic recombination on the selection analysis, a modified topology of ML trees was applied to the selection analysis by identifying non-recombinant regions and allowing each to have its phylogenetic tree. The modified and fitted trees were obtained by using the GARD (http://www.datamonkey.org/gard) (Kosakovsky Pond et al., 2006; Pond & Frost, 2005), which can screen an alignment for recombination breakpoints, infer a unique phylogenetic history for each detected recombination block, and generate a modified tree topology. The HyPhy software package was also employed to validate the results obtained using the ML method (Kosakovsky Pond et al., 2019). Fixed Effects Likelihood (FEL), Fast, Unconstrained Bayesian AppRoximation (FUBAR), Evolutionary Fingerprinting, and Mixed Effects Model of Evolution (MEME) algorithms were used (Kosakovsky Pond & Frost, 2005; Murrell et al., 2013). These methods can take recombination into account by screening recombination breakpoints of the sequences, identifying non-recombinant regions and allowing each to have its own phylogenetic tree by using an updated partitioned dataset provided by GARD (Pond et al., 2006).

### Mapping of positively selected sites to structure models of proteins

The three-dimensional structure of SidJ and CaM was modeled using the Phyre server (Kelley et al., 2015), and the SWISS-MODEL (http://swissmodel.expasy.org) (Waterhouse et al., 2018). The positive selection sites were mapped onto the structure and visualized by PyMol (http://www.pymol.org/) (Lilkova et al., 2015).

**Figure 1:**
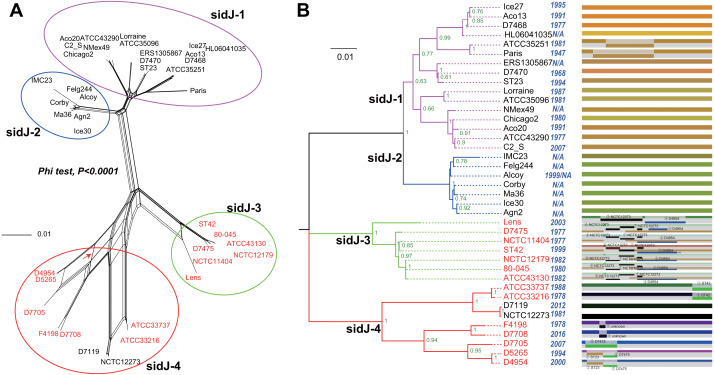
Phylogenetic relationships among *L. pneumophila sidJ* alleles. (A) Neighbor-net phylogenetic network shows the relationship among 39 *sidJ* alleles (see Table S1). The sidJ-1 subgroup is shown in a purple cycle, sidJ-2 blue, sidJ-3 green, and sidJ-4 red. The internal nodes represent hypothetical ancestral alleles and edges correspond to reticulate events such as recombination in the evolution of *sidJ*. The red arrow points to a representative reticulate event. (B) The evolutionary history of *sidJ* was inferred by using the Maximum Likelihood method. The ML tree was constructed from the alignment of nucleotide sequences of 39 alleles. Allele names were marked as their representative strain names. The earliest possible year for the allele to arose is shown in blue. Numbers on the interior branches represent bootstrap values and are indicated when the values are >0.5. The tree is drawn to scale, with branch lengths measuring in the number of substitutions per site. Branches in the same color were clustered into a group. Allele names are marked in red to indicate that they are recombinants. Unique recombination events detected by six recombination detection methods implemented under the RDP4 based on *sidJ* alleles are mapped onto the corresponding breaking point positions in the alignment (in the right of the figure). Recombination events that were identified by four or more methods were selected and numbered according to the RDP4 analysis, and the minor parent names of the recombinant alleles are shown nearby the breaking point positions (see Table 1).

## Results and Discussion

### Characteristics of *L.pneumophila* strains and *sidJ* sequences

The *sidJ* sequences of the 116 *L. pneumophila* strains in this study could be clustered into 39 unique alleles (alleles were marked with representative strain name, shown in Table S1), which corresponded to 36 different SidJ amino acid sequences*.* We propose that these sequences might represent most of the *sidJ* alleles. A total of 544 polymorphic (segregating) sites in the 39 *sidJ* alleles (the full-length gene is 2,622 bp) generate high amino acid sequence polymorphism in the SidJ protein, about one fifth (19.68%, 172/874) amino acid sites were polymorphic. Significantly higher amino acid sequence polymorphisms were found in the NTD than the CTD (24.78% Vs. 15.35%, 83/335 Vs. 43/280, Chi-Square test, *P* = 0.019).

### Intragenic recombination drives the evolution of *sidJ*

A reticulate network tree was obtained by the Neighbor-net algorithm of SplitsTree4, using the alignment of the 39 *sidJ* alleles. As shown in Fig. 1A, we could observe many reticulate events which indicated possible recombination events among *sidJ* alleles. Moreover, the implemented Phi test in SplitsTree4 did find significant evidence for recombination within the alleles (*P* < 0.001). Thus, we tested the intragenic recombination by using RDP 4. Eight potential recombination events (PREs) and 14 recombinant alleles were identified, which were supported by at least four of the six analysis methods according to Costa et al. (2014) report (Table 1). Figure 1B showed the phylogenetic relationship of these alleles. Allele names were shown with their representative strain names. Four main clades were found and all recombinants *sidJ* were distributed in clades three and four. Two non-recombinant *sidJ* alleles, D7119 and NCTC12273 formed a sub-clade and had a relatively far relationship with those non-recombinants. We observed that the stains harboring such alleles (D7119 and NCTC12273) all belonged to *L. pneumophila subsp. pascullei* and most were from environmental sources. In contrast, three recombinant *sidJ* alleles, D5265, D4954, and D7705 also formed a sub-clade but stains harbored such alleles all belonged to *L. pneumophila subsp. raphaeli* and most were from clinical sources. Previously reports by Costa et al. (2014) indicated that intragenic recombination was an important strategy in the evolutionary adaptive process of *sidJ*. We here showed similar results, but due to the fact that in this study a greater number of *L. pneumophila* strains and alleles of sidJ were used, we can consider our results more robust.

**Table 1:** Intragenic recombination in the 39 allelles of *sidJ* by using six different methods implemented in RDP software.

**Recombination events**	**Recombinant** **alleles**	**Major parent^#^**	**Minor parent^$^**	**Detection methods implemented in RDP software^∧^**					
				**RDP**	**GENECONV**	**Bootscan**	**Maxchi**	**Chimaera**	**SiSscan**
1	ATCC33216, ATCC33737	NCTC12273	ST42	N^a^	Y^b^	N	Y	Y	Y
2	D4954, D5265	D7705	ST23	Y	Y	Y	Y	Y	Y
3	Lens	Fleg244	NCTC12273	Y	Y	N	Y	Y	Y
4	Lens, 80-045, ATCC43130, NCTC12179, D7475, ST42, NCTC11404	Lorriane	D4954	N	Y	N	Y	Y	Y
5	D7705, D5265, D4954	NCTC12273	D7475	Y	Y	Y	Y	Y	Y
6	D7475, ATCC43130, NCTC12179, ST42, 80-045, NCTC11404	NMex4	NCTC12273	Y	Y	N	Y	Y	Y
7	ATCC43130, NCTC12179, D7475, ST42, 80-045, NCTC11404	Aco20	NCTC12273	Y	Y	N	Y	N	Y
8	F4198, D7708	ATCC35251	Unknown	Y	Y	Y	Y	Y	N

**Notes.**

* The allele names are shown as their representative strain’ names.

∧ Recombination events detected by more than three methods are shown.

# Major parent: parent sequences contribute the larger fraction of the sequence.

$ Minor parent: parent sequences contribute the smaller fraction of the sequence.

a N indicates recombination events were not detected by the method.

b Y indicates recombination events were detected by the method.

### Intragenic recombination drives the diversification of the *sidJ*

**Figure 2:**
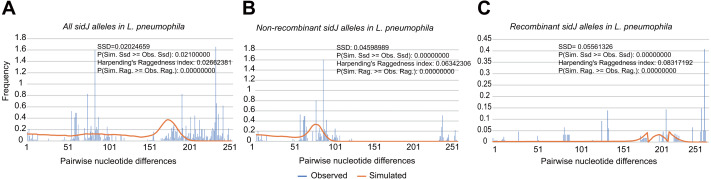
Frequency distribution of the number of pairwise nucleotide differences (mismatch) between *sidJ* alleles (haplotypes). (A) Mismatch distribution for the total data set (39 alleles); and the two allele groups including the (B) non-recombinant alleles and the (C) recombinant alleles are indicated. The solid orange line is the theoretical distribution under the assumption of population expansion.

**Figure 3:**
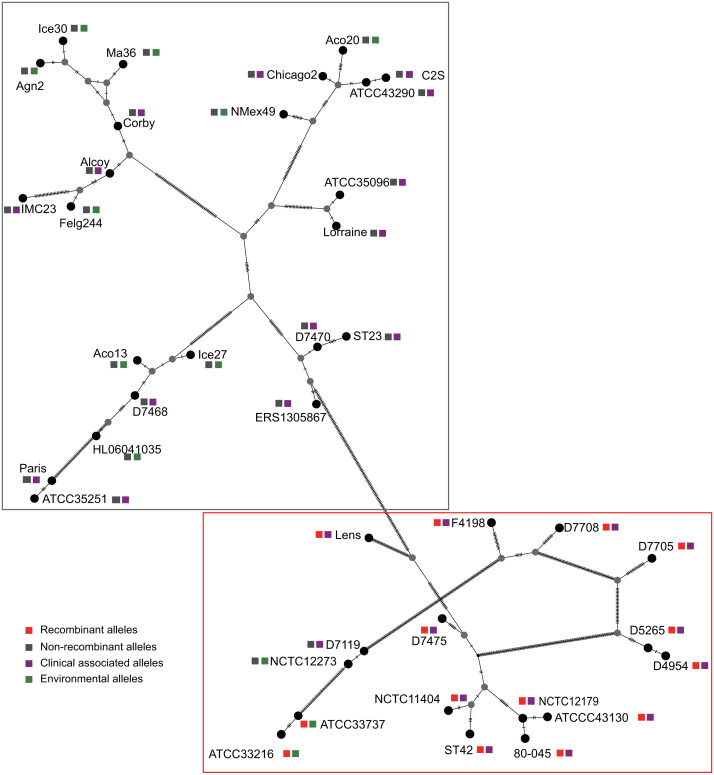
Parsimony (TCS) network of *L. pneumophila* sidJ haplotypes (alleles). The alleles were obtained from 116 worldwide isolates. Each oblique line between haplotypes (haplotype name is shown as its representative isolate name) represents one mutational difference. The connections are mutational steps between individuals. Unlabeled nodes (gray circles) indicate inferred steps that have not been found in the sampled populations as of yet. Boxes indicate major haplotype groups. Most haplotypes included in red dotted boxes are the recombinant ones, while those included in gray boxes are non-recombinant ones.

To explore whether intragenic recombination drives the diversification of the *sidJ,* we categorized the 116 strains into two groups: the recombinant group and the non-recombinant group. Then, we utilized DnaSP to study the difference in genetic diversity between the two groups. It showed that most of the parameters that represent genetic diversity were higher in the recombinant group. These parameters included nucleotide diversity, polymorphic sites, and the average number of nucleotide differences (Table 2), suggesting that recombination added a high density of polymorphisms in *sidJ*. The phylogeny of *sidJ* alleles also showed that recombinant alleles roughly formed an outside subclade (sidJ-3 and sidJ-4, Fig. 1B) compared with those non-recombinants. Recombination also introduced an excess of non-synonymous and synonymous diversity for *sidJ* (0.02454 Vs. 0.01371 and 0.1660 Vs.0.1063, Table 2). These results were partly consistent with the research with another intracellular bacteria, *Mycobacterium tuberculosis,* of which the genetic diversification was partly driven by recombination and led to a high genetic diversity and genomic plasticity (Namouchi et al., 2012). Furthermore, we did not find evidence for recombinant *sidJ* alleles experiencing demographic expansion based on the current pools of strains. The mismatch distributions for the total *sidJ* set, recombinant *sidJ*, and non-recombinant *sidJ* genes were roughly multimodal with *P* > 0.05 for the SSD. Also, *P*-values for Harpending’s Raggedness index was lower than 0.05 in each group, indicating that no demographic expansion exists (Figs. 2A–2C). However, a potential reduction of recombinant *sidJ* alleles in the *L. pneumophila* community was found. Fu and Li’s D* and F* tests showed significantly positive values (Table 2), indicating an excess of intermediate-frequency alleles which might result from bottleneck populations, thus causing demographic reduction (Rowbotham, 1980). Parsimony (TCS) network of *sidJ* alleles showed no central allele (node). Many mutations were found among the alleles, and these alleles did not comprise a scattered star structure (Fig. 3), suggesting that the expansion of the *L. pneumophila* population with a specific mutation in the *sidJ* gene has not taken place (Leigh & Bryant, 2015).

**Table 2:** Summary of genetic diversity parameters for ***sidJ*from*L. pneumophila*** strains.

**Parameters**	**Over all**	**Non-recombinant alleles**	**Recombinant alleles**
Sequences, n	116	83	33
Haplotypes, h	39	25	14
Haplotype diversity, Hd	0.909	0.843	0.866
Nucleotide diversity, *π*	0.05317	0.03178	0.05123
(standard deviation)	0.00278	0.00342	0.00193
Polymorphic sites, S	544	390	361
Theta per site (from S)	0.03900	0.03178	0.03396
(standard deviation)	0.00925	0.00755	0.01029
Average number of nucleotide differences, k	139.246	83.255	134.163
Total number of mutations, Eta	600	414	381
*dN*	0.02344	0.01371	0.02454
*dS*	0.1833	0.1063	0.1660
*dN/dS*	0.1278	0.1290	0.1478
Tajima’s D	0.7917 (*P* > 0.10)	0.01120 (*P* > 0.10)	1.64769 (*P* > 0.10)
Fu and Li’s D*	1.46609 (0.10 > *P* > 0.05)	1.30041( *P* > 0.10)	1.62736 (*P* < 0.02)
Fu and Li’s F*	1.38900 (*P* > 0.10)	0.90431 (*P* > 0.10)	1.94078 (*P* < 0.02)

**Figure 4:**
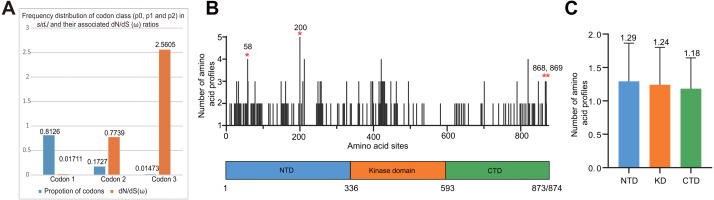
Frequency distribution of codon classes of *sidJ*, and domain architecture and amino acid profiles of each domain in the SidJ. (A) Frequency distribution of codon classes (p0 = negative selection, p1 = neutral selection, and p2 = positive selection) and their associated dN/dS (*ω*) ratios under M3 model. (B) Domain architecture of SidJ depicting the NTD (blue), the KD (orange), and the CTD (green). Amino acid profiles of each site are indicated. Positive selection sites are marked in red, and the number of their amino acid profiles is shown. (C) An average number of amino acid profiles in each domain. Data are shown in mean and standard deviation.

**Table 3:** Log-likelihood values and parameter estimates for the ***sidJ*gene of*L. pneumophila*using modifiedtopology tree of the alleles**.

Model	*nP*	*lnL*	Estimates of parameters	LRT *P-value*	Positively sites
M3 (discrete)	81	−9330.3176	p0 = 0.8126, p1 = 0.1727, p**2 = 0.01473**, *ω*0 = 0.01711, *ω*1 = 0.7739, *ω*2 = 2.5605	*P <10* ^−9^	200N*, 868T*, 869S**
M0 (one ratio)	77	−9567.0414	*ω*0 = 0.1636		Not Allowed
M2a (selection)	80	−9331.9663	p0 = 0.8393, p1 = 0.1533, **p2 = 0.00744**, *ω*0= 0.02473, *ω*1 = 1.00000, ***ω*2= 3.1041**	*P*=*0.0803*	**N/A**
M1a (neutral)	78	−9334.4879	p0 = 0.8392, p1 = 0.1608 *ω*0= 0.02390, *ω*1= 1.0000		Not Allowed
M8^a^ (beta& *ω*)	80	−9333.3117	p0 = 0.8654, *p* = 0.04277, *q* = 0.3594 **p1 = 0.01345,***ω* = 1.1155	*P <10* ^−9^	58G**, 200N**, 820A*, 867R*, 868T**, 869S**
M7 (beta)	78	−9358.5621	*p* = 0.03809, *q* = 0.17020		Not Allowed

**Notes.**

*P* is the number of parameters in the *ω* distribution; lnL is the log likelihood; *ω* is ratio of *dN*/*dS*, LRT *P*-value indicates the value of chi-square test; Parameters indicating positive selection are presented in bold; positive selection sites were identified by the Bayes empirical Bayes (BEB) methods under M8 model or by Naive Empirical Bayes (NEB) methods under M3 and M2a models.

The posterior probabilities (*p*) ≥0.90, (*p*) ≥0.95 and *p* ≥ 0.99 are indicated by *, ** and ***, respectively. (Yang, 2007; Yang & Bielawski, 2000) recommended that results from M8 model were preferred to find sites under positive selection pressure, and it is more robust to recombination which was proved by Anisimova, Nielsen & Yang (2003)

### Evidence of positive selection in *sidJ*

The M0 model of the EasyCodeML package showed an average *ω* of 0.1636 which was less than 1, suggesting that at the whole gene level, purifying selection conducting the evolution of *sidJ*. Moreover, the frequency distribution of codon classes based on the M3 model showed proportion of *sidJ* codons that was subject to purifying selection was 0.8176 (Fig. 4A), further proved that purifying selection was a major force during *sidJ* evolution. Although the proportion of codon 3 (under positive selection) was relatively smaller (0.01473, Fig. 4A), the three likelihood ratio tests (LRTs) showed that model M3 and M8 were significantly better fit (*P* < 0.05) than the relevant null model M0 and M7, respectively. These results together suggested that a small number of codons of *sidJ* were subjected to positive selection pressure (*ω* =1.1155–2.5605). Here, we took results from models M7 Vs. M8 as a standard as Yang et al. recommended (Yang, 2007; Yang & Bielawski, 2000). Thus, four positive selection sites including 58G, 200N, 868T, and 869S were identified with posterior probabilities (Pr) of at least 0.95 (Table 3). Still, only eight PREs were identified among the 39 alleles, the PREs and allele sequences’ ratio was about 20%, indicating that the LRT was robustness to such low levels of recombination (<30%) (Anisimova, Nielsen & Yang, 2003). These nested models were also more realistic and showed more robust to recombination (Anisimova, Nielsen & Yang, 2003). Similar results were obtained when recombination was not taken into account by using the unmodified tree topology of the 39 *sidJ* alleles. 868T, a definitive positive selection site with Pr =0.973, when using the modified tree topology, was identified as a critical positive selection site (Pr =0.942). (Table S2). To further confirm our results, three additional algorithms implemented in the HyPhy software package were used to identify positive selection sites of SidJ. We could identify all of the same positive selection codons as we obtained from the codeML package (Table S3). Thus, combined with the results from different algorithms, finally, four sites including 58G, 200N, 868T, and 869S were identified as definitive positive selection sites of SidJ. These sites were distributed in either the NTD or CTD of SidJ (Fig. 4B)*.* The distributions of single amino acid polymorphic loci in the KD *versus* the NTD and CTD were not uneven (17.83% Vs. 20.45%, 46/258 Vs. 126/616, *P* =*0.464*, Chi-Square test), and average numbers of amino acid site profiles in each domain of SidJ were roughly the same (Fig. 4C)*.* This result further verified that positive selection pressure selectively operated on the NTD and CTD domains, but not on the KD. Based on this result, we propose that the KD domain of SidJ is essential, but conserved to maintain its glutamylase activity to catalyze the glutamylation of *L. pneumophila* SidEs, while NTD and CTD domains modulate the interaction between SidJ and CaMs from different hosts. Positive selection in the CTD and NTD of SidJ may be an evolutionary strategy for *L. pneumophila* surviving in different hosts. Few studies had focused on studying positive selection signals in the individual *L. pneumophia* genes. Kenzaka et al. (2018) previously reported preferential positive selection in F-Box Domain gene (*lpp0233*), and they found a higher *ω* in this gene compared with those in other protein encoding genes and housekeeping genes. However, the *ω* in *lpp0233* was less than 1 (0.40), indicating that at the whole gene level, the positive selection signal was still weak and codon level analysis was required. Costa et al. (2014) also attempted to discover positive selection sites of *sidJ* by using the M8 model but failed because the likelihood ratio tests between M8 and M7 models showed no significant difference. This might be due to the limited *sidJ* alleles (23 alleles) they obtained, as the codeML algorithm was phylogeny-based, and more allelic profiles of a gene could infer an authentic phylogenetic history much better. This result highlighted the importance of using enough alleles of a gene for analyzing positive selection at the codon level. We previously verified 14 positive selection sites of a key protein associated with an antibiotic-resistance characteristic of methicillin-resistant *Staphylococcus aureus,* named penicillin-binding protein (PBP) 2a (Zhan & Zhu, 2018). We found that all these sites in PBP2a have only one mutation profile. However, SidJ positive selection sites showed there were more mutation profiles in a codon. Three mutation profiles including G58R, G58M, and G58E were identified at codon 58. Four mutation profiles including N200T, N200I, N200A, and N200V were identified at codon 200. Two mutation profiles including T868N, T868P, and S869T, S869P were identified at codons 868 and 869, respectively (Table S4). Most of these mutation profiles included changes in chemical properties of the amino acids (*e.g.*, amino acid polarity) (Table S4), which might affect the three-dimensional structure of SidJ. SidJ modulates host cellular pathways through the membrane remodeling of *L. pneumophila* (Luo, 2012)*.* Given that SidJ interacts with both SdeA and eukaryotic CaM, diversified mutation profiles of these positive selection sites might imply that *sidJ* was a target for host specialization or selection and these mutations might increase the fitness of *L. pneumophila* in certain environments, and in turn promote their survival in different hosts (Costa et al., 2014; Park, Ghosh & O’Connor, 2020). However, the exact function of these mutations requires further study.

### Recombination and positive selection shape the population structure of SidJ in *L. pneumophila*

**Figure 5:**
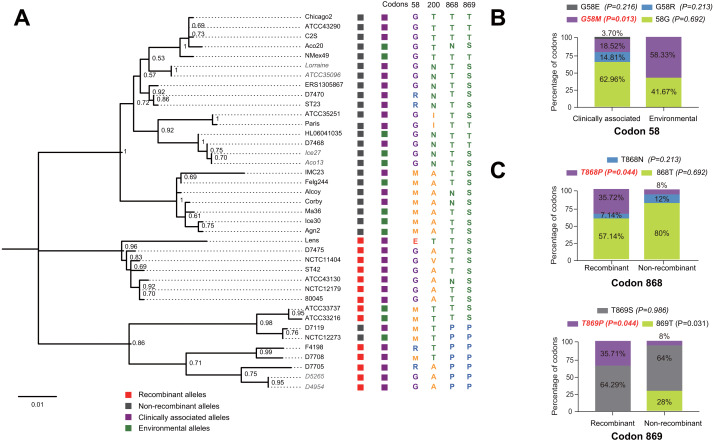
Phylogenetic relationships among SidJ** proteins of *L. pneumophila* from different sources, and with different mutation profiles at positive selection sites. (A) Numbers on the interior branches represent bootstrap values and are indicated when the values are > 0.5. Protein names were marked as their representative allele (strain) names. Alleles with the same protein sequences were marked in gray. (B) Composition of different mutation profiles of positive selection sites in the clinically associated and environmental SidJ, or (C) in the recombinants and non-recombinants. Comparison of frequencies of codons between different groups was carried out by using the Chi-square tests or Fisher’s Exact tests.

The evolutionary history of the SidJ proteins corresponding to 39 alleles was studied by using MEGA X. Similar topology of the trees was found when compared to that of the *sidJ* alleles (Figs. 1B and 4). Three paired alleles (Lorraine and ATCC35096, Ice27 and Aco13, and D5265 and D4954) encoded SidJ with the same protein sequences. The properties of these alleles and the information of their representative strains were studied. Of the 39 *sidJ* alleles, some were distributed both in clinical and environmental strains, while some were only distributed in environmental strains (Table S1). We defined those alleles that could be found in clinical strains as clinically associated alleles. Among the 14 recombinant alleles, 12 (85.71%) were clinically associated. In contrast, among the 25 non-recombinant alleles, only 15 (60%) were clinically associated. This result suggested that recombinant *sidJ* alleles were more likely to be clinically associated alleles, although not significant (*P* =*0.093*, Chi-Square test). (Fig. 5A). A larger pool of *L. pneumophila* strains is required to sufficiently explain the association of recombinant *sidJ* with clinical strain. Based on this result, we propose that recombination is an important strategy for *L. pneumophila* to survive in different environments, and for infecting human cells. We did find specific mutation profiles of positive selection sites in clinically associated alleles (Fig. 5B) or recombinant alleles (Fig. 5C), for example G58M mutation happened less frequently in clinically associated alleles, while T868P, T869P happen more frequently in recombinant alleles of *sidJ* (Table S5 and Figs. 5B–5C). Considering that the positive selection is usually beneficial to the survival of the individual bacteria carrying the mutation, these results indicated that mutations in positive selection sites increased SidJ variability, and made more extensive the adaptability in environmental hosts for *L. pneumophila.* This might lead to broad coevolution of *L. pneumophila* genes (*e.g.*, *sidJ*) with viable environmental hosts before it could infect human cells and thus be of crucial importance in the virulence of this bacteria. The fact that the finding of alleles harboring particular mutations in positive selection sites were more likely recombinants further demonstrated that recombination in *sidJ* enhanced the environmental adaptability of some *L. pneumophila* strains (Fig. 5C).

**Figure 6:**
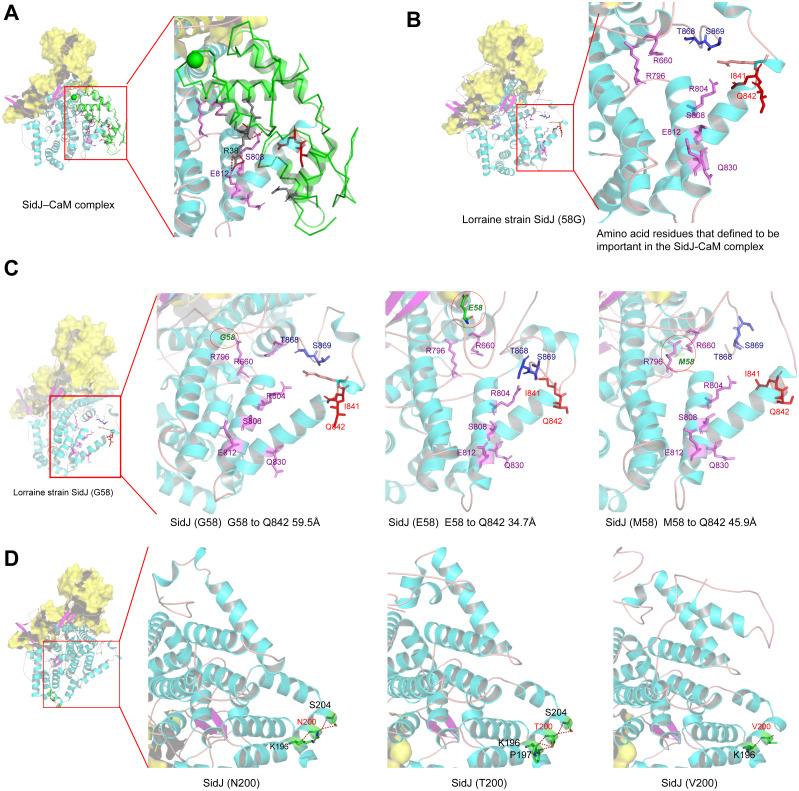
Structure of SidJ and potential influence of mutation in positive selection sites. The yellow covering shows the surface of the KD domain of SidJ. The secondary structure elements of SidJ are colored in cyan (helix), purple (sheet), and orange (loop) respectively. (A) Structure of the SidJ-CaM complex (PDB ID: 6K4K). The CaM is shown in green. The amino acid residues are shown as sticks. Purple sticks indicate a part of representative amino acid residues of SidJ that interact with CaM amino acid residues (gray sticks). Red sticks indicate the IQ motif (I841Q842) of SidJ. Hydrogen bonds (colored in red) including S808(SidJ) and E812(SidJ): R38(CaM) are shown. (B) The overall structure of the Lorraine strain SidJ. The relative position of amino acid residues that are defined to be important in interacting with CaM residues are shown as purple sticks and the IQ motif (I841Q842) is shown in red. (C) Mutation of positive selection site (codon 58) causes a distance change of closest atoms between the core of IQ motif which is crucial in SidJ-CaM binding. (D) The number of hydrogen bonds (colored in red) vary among different mutation profiles of codon 200.

**Figure 7:**
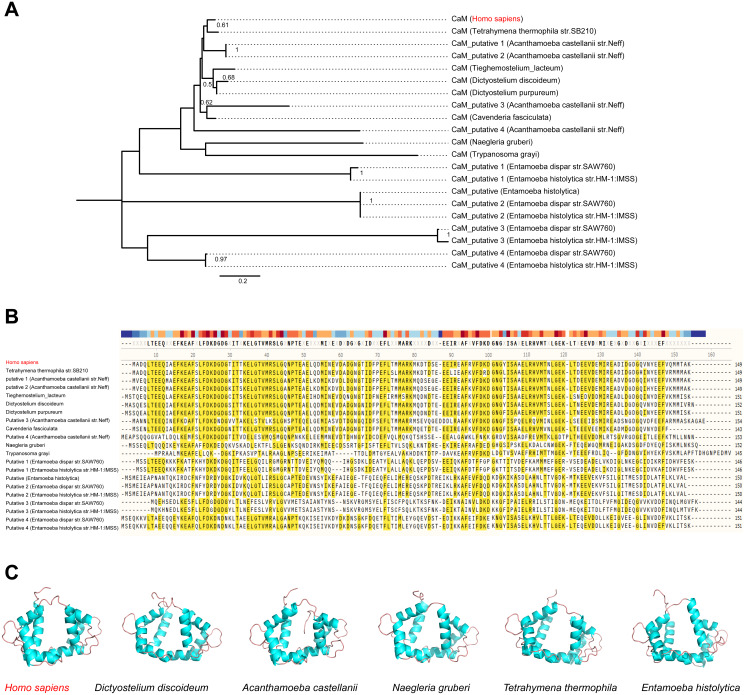
CaM is variable among potential *L. penumophila* hosts. (A) Phylogenetic relationship among CaM from *Homo sapiens* and other potential*****L. penumophila* environmental hosts. (B) Variability of CaM protein sequences among *Homo sapiens* and other potential *L. penumophila* environmental hosts. Amino acid residues marked in red indicate that 50% of the protein harbor the same residue in this site. (C) CaM protein structure comparisons among six potential *L. penumophila* hosts. The evolutionary history of CaM protein was inferred by using the Maximum Likelihood method and LG model. Bootstrap values were estimated using 1,000 replications. Numbers on the interior branches represent bootstrap values and are indicated when the values are > 0.5. Details of CaM from *Homo sapiens* and other potential*L. penumophila* environmental hosts are shown in Table S6. We utilized the CaM protein with the NCBI accession number AAD45181.1 as the representative for *Homo Sapiens*, AAA33172.1 as the representative for *Dictyostlium discoideum*, XP_004334690.1 as the representative for *Acanthamoeba castellanii*, XP_002674748.1 as the representative for *Naegleria gruberi*, XP_001022775.2 as the representative for *Tetrahymena thermophila*, and XP_651708.1 as the representative for *Entamoeba histolytica*. All three-dimensional structures of CaM are shown in the same visual angle.

## Mutation of positive selection sites of SidJ might influence the binding of CaM to SidJ

Here, the four definitive positive selection sites are located in the NTD and CTD, but not in the KD. CaM binding is required by SidJ glutamylase activity. An IQ (I841 and Q842) motif located in the C-terminal domain of SidJ is involved in CaM binding by burying in a hydrophobic cleft of the CaM C lobe. The CTD of CaM semi-encircles the C-terminal helix of SidJ and the NTD domain of CaM makes extensive contacts with the N-terminus of SidJ (Bhogaraju et al., 2019). Residues in these domains also play roles in mediating the formation of the SidJ-CaM complex, and in turn, stabilize the position of the N-lobe of the KD, and thereby leading to the formation of a stable catalytic pocket for SidES (Bhogaraju et al., 2019). A previous site-directed mutagenesis study verified the importance of I841 and Q842 for optimal binding. In addition, some other sites including Q830, S808, E812, R796, R660, R804 that engaged in hydrogen-bonding interactions with corresponding CaM residues also showed the importance of the binding affinity of SidJ with CaM (Gan et al., 2019). Therefore, the mutation on the positive selection sites might change the level of interactions of SidJ with CaM through hydrogen bonds and salt bridges. Structural studies on a truncated SidJ–CaM complex indicated that some of the residues in the C- terminus of SidJ were crucial to the complex which is adjacent to the IQ motif (Fig. 6A). Our protein structural modeling showed a similar three-dimensional image as the truncated one (Fig. 6B). The definitive positive selection site on codon 58 of SidJ indicated that the mutation of this site was functional, although a previous study suggested that a truncated SidJ lacking the first 99 residues (SidJ(ΔN99)) showed activity indistinguishable from that of the full-length protein (Gan et al., 2019). This implied that a full-length protein of SidJ was required for a more detailed description of the function of some important amino acid sites of the whole protein. As shown in Fig. 6C, the G58E or G58M mutations significantly influence the three-dimensional structure of SidJ. The G58E mutation might be associated with the interaction with CaM because it was spatially closer to the I841 and Q842 than the original 58G (Fig. 6C). A similar explanation of the influence of codons 868 and 869 was determined as they were closer to those that could interact with CaM residues (Fig. 6B) and where the CaM docked and could mediate most of the interactions with CaM (Bhogaraju et al., 2019). Although codon 200 of SidJ had four mutation profiles, we found two of which might be functional. As shown in Fig. 6D, the N200T and N200V mutations significantly affected the hydrogen bonds of SidJ. Two hydrogen bonds of N200 could be formed with K196 and S204, while T200 could form three ones with K196, P197, and S204. In contrast, V200 could only form one with K196 (Fig. 6D). The more hydrogen bonds indicated a more stable *α*-helix, in turn, stabilizes the whole protein structure. Thus, the mutations in codon 200 may also mediate the interaction between SidJ and CaM and all these mutations in the positive selection sites could only adjust, but not abolish the affinity of CaM binding to SidJ. Based on protein structure modeling, we propose a potential explanation of the influence of the mutation on the four positive selection sites. Experimental data was still required to understand the exact function of these mutations. Mutation in positive selection sites might facilitate the survival of the lifeform containing mutated alleles. As an intracellular bacterium, entering the host and establishing infection were crucial for *L. pneumophila* lifecycle, and in which SidJ was dedicated to balance the host Ub ligase activity and was important for successful infection. *L. pneumophila* was shown to survive as an intracellular parasite of free-living protozoa in aquatic and moist soil environments (Bhogaraju et al., 2019; Fields, Benson & Besser, 2002; Gan et al., 2019). Protozoa provide a specific environment for gene exchange between *L. pneumophila* and other microorganisms invading them as pathogens or symbionts, also protozoa might be act as donors and transfer their own DNA to *L. pneumophila* (Mondino, Schmidt & Buchrieser, 2020). Many potential environmental hosts of *L. pneumophila* have been identified, including *Dictyostelium discoideum* (soil amoeba), *Acanthamoebae castellanii*, *Entamoeba histolytica*, *Naegleria,* and *Tetrahymena*, etc*.* (Hagele et al., 2000; Steinert et al., 1994; Rowbotham, 1980; Swart et al., 2018). Figure 7A showed the phylogenetic relationship of CaM among humans and potential hosts of *L. pneumophila*. Despite that CaM was relatively conserved, the protein sequences of CaM in these eukaryotes are variable (Fig. 7B, Table S6). And this might lead to a slightly structural difference among human CaM and those of protozoa hosts of *L. pneumophila* (Fig. 7C). Given that most of the time *L. pneumophila* is inhabiting in environmental hosts, we also propose that the variability in SidJ, especially that in positive selection sites might be important towards *L. pneumophila* survival in different environmental hosts and has an adaptive function to a broad selection of environments. The exact functions of these mutations to *L. pneumophila* living in the environmental host are worthy of further study. These results also suggested that more natural variants of a protein from a broad-host bacterium were required to discover the mechanism of how this protein and its variants are involved in the infection.

## Conclusions

We presented molecular evolution analyses on a large and comparative set of *sidJ* alleles, derived from a collection of *L. pneumophila* strains. We found that among the 39 recognized *sidJ* alleles, about one-third were recombinants generated by eight PREs. Intragenic recombination also drove the *sidJ* diversification manifested by a higher genetic diversity in the recombinants as compared with that in non-recombinants. In addition, we found definitive positive Darwinian selection of SidJ at the codon level. Four codons in the NTD and CTD domains of SidJ were identified, and their mutation profiles were also determined. Protein structural modeling of SidJ provided possible functional explanations for the mutations in positive selection sites. It might influence the binding affinity of CaM to SidJ, thus regulate SidJ glutamylase activity to SidEs, and in turn balance the Ub ligase activity in different hosts. This study gave us a deeper understanding of the adaptive mechanisms of this intracellular bacterium to different hosts and highlighted the importance of the NTD and CTD domains in SidJ kinase activity that is activated by the binding of CaM. Further studies should focus on experimental evidence to illustrate the function and mechanism of natural *sidJ* mutants (with a mutation in positive selection sites) in regulating balanced Ub ligase activity in different *L. pneumophila* hosts.

##  Supplemental Information

10.7717/peerj.12000/supp-1 Supplemental Information 1 Information of the 116 *L. pneumophila* strains *N/A indicates not available. #Strain or sample names marked red indicate representative allele names.

10.7717/peerj.12000/supp-2 Supplemental Information 2 Log-likelihood values and parameter estimates for the *sidJ* gene of *L. pneumophila* using unmodified topology tree of the alleles. *P* is the number of parameters in the *ω* distribution; lnL is the log likelihood; *ω* is ratio of *dN*/*dS*, LRT *P*-value indicates the value of chi-square test; Parameters indicating positive selection are presented in bold; positive selection sites were identified by the Bayes empirical Bayes (BEB) methods under M8 model or by Naive Empirical Bayes (NEB) methods under M3 and M2a models. The posterior probabilities (*p*) ≥ 0.90, (*p*) ≥ 0.95 and *p* ≥ 0.99 are indicated by *, ** and ***, respectively. Yang et al. recommended that results from M8 model were preferred to find sites under positive selection pressure, and it is more robust to recombination which was proved by Maria et al.

10.7717/peerj.12000/supp-3 Supplemental Information 3 Parameter estimates for the *sidJ* gene of *L.pneumophil* a and positive selection sites detected by methods implemented in HyPhy package *α* indicates(dS) value and *β* or *β*+ indicates(dN) values. # Cut-off value for *P*-values in MEMER was set at 0.03 to enhance the analysis specificity . *Cut-off value for Posterior Pr in FUBAR was set at 0.90.

10.7717/peerj.12000/supp-4 Supplemental Information 4 Mutation profiles of positive selection sites in SidJ PNA: *P* olar *n* eutral *a* mino acidPAA: *P* olar *a* cidic *a* mino acidPBA: *P* olar *b* asic *a* mino acidNPA: Non-*p* olar *a* mino acid*Underlines indicate that these mutation profiles have corresponding amino acid substitution model.

10.7717/peerj.12000/supp-5 Supplemental Information 5 Positive selection sites codon distribution patterns in different types of alleles * Fisher’s exact test was used for comparing distribution differences of mutation profiles in different types of *sidJ* alleles. # The red color indicates mutation profiles have distinct distribution between different types of *sidJ* alleles.

10.7717/peerj.12000/supp-6 Supplemental Information 6 Information of CaM protein in different species of potential *L. pneumophila* hosts

10.7717/peerj.12000/supp-7 Supplemental Information 7 SidJ gene and protein sequences obatined from NCBI database igase activity in different *L. pneumophila* hosts.

## Additional Information and Declarations

### Data Availability

The following information was supplied regarding data availability: The strain information including strain name and GenBank accession number is available in the Supplemental Files, as are the gene and protein sequences of SidJ from the NCBI database.
