# Supplementary material for: Virulence effector SidJ evolution in Legionella pneumophila is driven by positive selection and intragenic recombination
Source: PeerJ. 2021 Aug 17;9:e12000. doi: 10.7717/peerj.12000 (PMC8378335; doi:10.7717/peerj.12000)
Supplement: Supplemental Information 1 — *N/A indicates not available. #Strain or sample names marked red indicate representative allele names. [file peerj-09-12000-s001.docx]

**Table S1. Information of the 116 *L. pneumophila* strains.**

| Strain Name/ Allele name | Serogroup | Subspecies | Source nature | Geographic location | Collection year | NCBI BioSample No. | Accession No. | *sidJ* location | Reference |
| --- | --- | --- | --- | --- | --- | --- | --- | --- | --- |
| ***sidJ* alleles that are not recombinants** |  |  |  |  |  |  |  |  |  |
| **Allele 1** |  |  |  |  |  |  |  |  |  |
| ***Lorraine#*** | 1 | *pneumophila* | Clinical | France: Lorraine | 2002 | SAMEA3138425 | FQ958210 | 2311518-2314139 | [1] |
| ERS1211138 | 8 | *pneumophila* | Clinical | N/A | 1987 | SAMEA4040028 | LT906452 | 2443416-2446037 | N/A |
| **Allele 2** |  |  |  |  |  |  |  |  |  |
| ***ATCC35096*** | 8 | *pneumophila* | Clinical | USA: California, Concord | 1981 | SAMN03481609 | HG531940 | 1-2625 | [2] |
| **Allele 3** |  |  |  |  |  |  |  |  |  |
| Birmingham 1 (D-7470) | 1 | *pneumophila* | Clinical | USA: Alabama, Birmingham | 1977 | SAMN06928756 | CP021268 | 2395759-2398383 | [3] |
| ST62 | 1 | *N/A* | Clinical | N/A | 2004 | SAMEA4535100 | LT632614 | 2456117-2458741 | N/A |
| Pontiac | 1 | *N/A* | Environmental | USA: Pontiac, Michigan | 1968 | SAMN05199028 | CP016029 | 2456510-2459134 | N/A |
| L10-023 | 1 | *N/A* | Clinical | Germany | 2010 | SAMN03379010 | CP011105 | 2440890-2443514 | [4] |
| **Allele 4** |  |  |  |  |  |  |  |  |  |
| ***ERS1305867*** | N/A | *N/A* | Clinical | N/A | N/A | SAMEA4394418 | CP048618 | 2394643-2397267 | N/A |
| Toronto-2005 | N/A | *pneumophila* | Clinical | Canada: Toronto | 2005 | SAMN03839261 | CP012019 | 2410891-2413515 | [5] |
| **Allele 5** |  |  |  |  |  |  |  |  |  |
| ***ST23*** | 1 | *N/A* | Clinical | France | 1994 | SAMEA4535097 | LT632615 | 2440067-2442691 | [6] |
| **Allele 6** |  |  |  |  |  |  |  |  |  |
| ***Chicago2*** | 6 | *pneumophila* | Clinical | N/A | 1980 | N/A | HG531934 | 1-2625 | [2] |
| **Allele 7** |  |  |  |  |  |  |  |  |  |
| SBT211 | N/A | *N/A* | Clinical | Australia | 2016 | SAMN13191634 | CP045974 | 2458506-2461127 | N/A |
| NCTC12180 | 12 | *pneumophila* | Clinical | USA | 1986 | SAMEA3111980 | LR133933 | 2379102-2381723 | [7] |
| Burlington 1 (D-7841) | 1 | *pneumophila* | Clinical | USA: Vermont, Burlington | 1977 | SAMN06928757 | CP021267 | 2371340-2373964 | N/A |
| Philadelphia-1 | 1 | *pneumophila* | Clinical | USA:Philadelphia, Pennsylvania | 1976 | SAMN03988484 | CP013742 | 2418025-2420646 | N/A |
| ST37 | 1 | *N/A* | Clinical | UK | 2003 | SAMEA4535098 | LT632616 | 2484328-2486949 | [8] |
| E9_O | N/A | *N/A* | Environmental | USA: Illinois | 2012 | SAMN05180041 | CP015956 | 2376831-2379452 | [9] |
| E8_O | N/A | *N/A* | Environmental | USA: Texas | 2006 | SAMN05180040 | CP015955 | 2408040-2410661 | [9] |
| E7_O | N/A | *N/A* | Environmental | USA: Georgia | 2009 | SAMN05180039 | CP015954 | 2380050-2382671 | [9] |
| E6_N | N/A | *N/A* | Environmental | USA: New Jersey | 2011 | SAMN05180038 | CP015953 | 2372312-2374933 | [9] |
| E5_N | N/A | *N/A* | Environmental | USA: Arkansas | 2011 | SAMN05180037 | CP015951 | 2373875-2376496 | [9] |
| E4_N | N/A | *N/A* | Environmental | USA: Alabama | 2012 | SAMN05180036 | CP015950 | 2372442-2375063 | [9] |
| E3_N | N/A | *N/A* | Environmental | USA: Texas | 2011 | SAMN05180035 | CP015949 | 2419410-2422031 | [9] |
| C11_O | N/A | *N/A* | Clinical | USA: Georgia | 2009 | SAMN05180032 | CP015945 | 2370925-2373546 | [9] |
| C10_S | N/A | *N/A* | Clinical | USA: Nebraska | 1990 | SAMN05180031 | CP015944 | 2378412-2381033 | [9] |
| C9_S | N/A | *N/A* | Clinical | USA: Indiana | 1982 | SAMN05180030 | CP015941 | 2371944-2374565 | [9] |
| C8_S | N/A | *N/A* | Clinical | USA: Colorado | 1993 | SAMN05180029 | CP015939 | 2374058-2376679 | [9] |
| C7_O | N/A | *N/A* | Clinical | USA: Delaware | 1994 | SAMN05180028 | CP015938 | 2369527-2372148 | [9] |
| C6_S | N/A | *N/A* | Clinical | USA: New Jersey | 1996 | SAMN05180027 | CP015937 | 2371336-2373957 | [9] |
| C5_P | N/A | *N/A* | Clinical | USA: Ohio | 1998 | SAMN05180026 | CP015936 | 2373786-2376407 | [9] |
| C4_S | N/A | *N/A* | Clinical | USA: Georgia | 2000 | SAMN05180025 | CP015935 | 2363430-2366051 | [9] |
| C3_O | N/A | *N/A* | Clinical | USA: Texas | 2006 | SAMN05180024 | CP015934 | 2405886-2408507 | [9] |
| Philadelphia_1_CDC | 1 | *N/A* | Clinical | USA:Pennsylvania | 1977 | SAMN05180044 | CP015928 | 2417444-2420065 | [9] |
| E11_U | N/A | *N/A* | Environmental | USA: Iowa | 2006 | SAMN05180043 | CP015926 | 2409936-24125577 | [9] |
| E10_P | N/A | *N/A* | Environmental | USA: Ohio | 2007 | SAMN05180042 | CP015925 | 2369629-2372250 | [9] |
| Lpm7613 | 1 | *N/A* | Clinical | Australia | 2000 | [SAMEA4067783](https://www.ncbi.nlm.nih.gov/biosample/SAMEA4067783/) | LT598657 | 2270058-2272679 | [10] |
| Thunder Bay | 6 | *pneumophila* | Clinical | Canada | N/A | SAMN02603729 | CP003730 | 2476744-2479365 | [11] |
| LPE509 | 1 | *pneumophila* | Environmental | N/A | N/A | [SAMN02604344](https://www.ncbi.nlm.nih.gov/biosample/SAMN02604344/) | CP003885 | 986266-988887 | [12] |
| ***ATCC43290*** | 12 | *pneumophila* | Clinical | USA:Colorado | 1986 | SAMN01917404 | CP003192 | 2369540-2372161 | [13] |
| Philadelphia 1 | 1 | *pneumophila* | Clinical | USA:Philadelphia | 1976 | SAMN02603156 | AE017354 | 2407667-2410288 | [14] |
| **Allele 8** |  |  |  |  |  |  |  |  |  |
| ***NMex49*** | N/A | *pneumophila* | Environmental | N/A | N/A | N/A | HG531948 | 1-2625 | [15] |
| **Allele 9** |  |  |  |  |  |  |  |  |  |
| ***C2_S*** | N/A | *N/A* | Clinical | USA: Illinois | 2007 | SAMN05179997 | CP015933 | 2371333-2373954 | [9] |
| **Allele 10** |  |  |  |  |  |  |  |  |  |
| ***Aco20*** | N/A | *pneumophila* | Environmental | Portugal | 1991 | N/A | HG531937 | 1-2625 | [15] |
| **Allele 11** |  |  |  |  |  |  |  |  |  |
| ***HL06041035*** | 1 | *pneumophila* | Environmental | N/A | N/A | SAMEA3138424 | FQ958211 | 2473122-2475746 | [16] |
| **Allele 12** |  |  |  |  |  |  |  |  |  |
| ***Ice27*** | 1 | *pneumophila* | Environmental | Iceland | 1995 | SAMEA769974 | HG531942 | 1-2625 | [15] |
| **Allele 13** |  |  |  |  |  |  |  |  |  |
| ***Aco13*** | N/A | *pneumophila* | Environmental | Portugal | 1991 | N/A | HG531936 | 1-2625 | [15] |
| **Allele 14** |  |  |  |  |  |  |  |  |  |
| ***D7468*** | 1 | *pneumophila* | Clinical | USA: Tennessee, Knoxville | 1977 | SAMN06928759 | CP021266 | 2369418-2372043 | [3] |
| ERS1211137 | 1 | *N/A* | Clinical | USA | 1980 | SAMEA4040027 | LT906476 | 2369418-2372043 | N/A |
| F4468 | N/A | *N/A* | Clinical | USA: New York | 2015 | SAMN04544730 | CP014759 | 2414562-2417186 | N/A |
| F4469 | N/A | *N/A* | N/A | USA:New York | 2015 | SAMN04544525 | CP014760 | 2414563-2417187 | N/A |
| D7630 | 1 | *N/A* | Clinical | USA: New York | 2012 | SAMN04634513 | CP015344 | 2423504-2426128 | N/A |
| D7631 | 1 | *N/A* | Environmental | USA: New York | 2012 | SAMN04634091 | CP015343 | 2412818-2415442 | N/A |
| D7632 | 1 | *N/A* | Clinical | USA: New York | 2012 | SAMN04633978 | CP015342 | 2412286-2414910 | N/A |
| **Allele 15** |  |  |  |  |  |  |  |  |  |
| ***ATCC35251*** | 15 | *fraseri* | Clinical | N/A | 1981 | SAMN02650973 | HG531945 | 1-2625 | N/A |
| **Allele 16** |  |  |  |  |  |  |  |  |  |
| FDAARGOS_779 | N/A | *N/A* | N/A | USA:VA | N/A | SAMN11056494 | CP040987 | 1347709-1350333 | N/A |
| NCTC11193 | 1 | *N/A* | N/A | N/A | 1977 | SAMEA3923587 | LR134332 | 2453659-2456283 | N/A |
| D7474 | 1 | *pneumophila* | Clinical | [USA: New Mexico, Albuquerque](https://www.ncbi.nlm.nih.gov/biosample?term=) | 1977 | SAMN06928753 | CP021286 | 2397070-2399694 | [3] |
| D7477 | 1 | *pneumophila* | Clinical | USA: Michigan, Flint | 1977 | SAMN06928758 | CP021281 | 2464638-2467262 | [3] |
| Lansing 3 | 15 | *fraseri* | Clinical | USA: Michigan, Royal Oak | 1981 | SAMN06928770 | CP021257 | 1016251-1018875 | N/A |
| E1_P | N/A | *N/A* | Environmental | USA: California | 2013 | SAMN05180033 | CP015946 | 2420179-2422803 | [9] |
| C1_S | N/A | *N/A* | Clinical | USA: North Carolina | 2009 | SAMN05179547 | CP015932 | 2458641-2461265 | [9] |
| Philadelphia_4 | 1 | *N/A* | Clinical | USA: Pennsylvania | 1977 | SAMN05180047 | CP015931 | 2455923-2458547 | [9] |
| Philadelphia_3 | 1 | *N/A* | Clinical | USA: Pennsylvania | 1977 | SAMN05180046 | CP015930 | 2418030-2420654 | [9] |
| Philadelphia_2 | 1 | *N/A* | Clinical | USA: Pennsylvania | 1977 | SAMN05180045 | CP015929 | 2455923-2458547 | [9] |
| Philadelphia_1_ATCC | 1 | *N/A* | Clinical | USA: Pennsylvania | 1977 | SAMN05180048 | CP015927 | 2455923-2458547 | [9] |
| OLDA | 1 | *N/A* | Clinical | Unknown | 1947 | SAMN05198688 | CP016030 | 2362727-2365351 | [3] |
| Por3 | 1 | *pneumophila* | Environmental | N/A | N/A | N/A | HG531949 | 1-2625 | [17] |
| ***Paris*** | 1 | *pneumophila* | Clinical | France: Paris | 1987 | SAMEA3138252 | CR628336 | 2378414-2381032 | [18] |
| **Allele 17** |  |  |  |  |  |  |  |  |  |
| NMex1 | 6 | *pneumophila* | Environmental | N/A | N/A | N/A | HG531947 | 1-2628 | [15] |
| **Alcoy** | 1 | *N/A* | Clinical | Spain: Alcoy | 1999 | SAMN02604292 | CP001828 | 2476952-2479579 | [19] |
| **Allele 18** |  |  |  |  |  |  |  |  |  |
| ***IMC23*** | 1 | *pneumophila* | Environmental | N/A | N/A | N/A | HG531944 | 1-2628 | [15] |
| **Allele 19** |  |  |  |  |  |  |  |  |  |
| Alf18 | N/A | *pneumophila* | Environmental | N/A | N/A | N/A | HG531939 | 1-2628 | [15] |
| ***Corby*** | 1 | *pneumophila* | Clinical | United Kingdom | N/A | SAMN02603241 | CP000675 | 2490944-2493571 | [20] |
| **Allele 20** |  |  |  |  |  |  |  |  |  |
| ***Felg244*** | N/A | *pneumophila* | Environmental | N/A | N/A | N/A | HG531941 | 1-2628 | [15] |
| **Allele 21** |  |  |  |  |  |  |  |  |  |
| ***Ma36*** | N/A | *pneumophila* | Environmental | N/A | N/A | N/A | HG531946 | 1-2628 | [15] |
| **Allele 22** |  |  |  |  |  |  |  |  |  |
| ***Ice30*** | N/A | *pneumophila* | Environmental | N/A | N/A | N/A | HG531943 | 1-2628 | [15] |
| **Allele 23** |  |  |  |  |  |  |  |  |  |
| ***Agn2*** | 1 | *pneumophila* | Environmental | Italy (Agano) | N/A | N/A | HG531938 | 1-2628 | [15] |
| **Allele 24** |  |  |  |  |  |  |  |  |  |
| F4185 | 1 | *pascullei* | Environmental | USA: Pennsylvania | 2012 | SAMN04438305 | CP014255 | 2349057-2351681 | [21] |
| ***D7119*** | 1 | *pascullei* | Clinical | USA: Pennsylvania | 2012 | SAMN04438273 | CP014257 | 2345263-2347887 | [21] |
| **Allele 25** |  |  |  |  |  |  |  |  |  |
| ***NCTC12273*** | N/A | *pascullei* | Environmental | USA | 1989 | SAMEA44537668 | LR134380 | 2370770-2373388 | N/A |
| NCTC12272 | N/A | *pascullei* | Environmental | USA | 1988 | SAMEA3905390 | LS483412 | 2370767-2373385 | N/A |
| D7160 | 5 | *pascullei* | Environmental | USA:Pennsylvania, Pittsburgh | 1981 | SAMN06928765 | CP021262 | 2370770-2373394 | [3] |
| D7158 | N/A | *pascullei* | Environmental | USA: Pennsylvania | 1982 | SAMN04438278 | CP014256 | 2370770-2373394 | [3] |
| ***sidJ* alleles are recombinants** |  |  |  |  |  |  |  |  |  |
| **Allele 26** |  |  |  |  |  |  |  |  |  |
| ***NCTC11404*** | 1 | *N/A* | Clinical | USA: Bellingham | 1981 | SAMEA3923592 | LS483410 | 2376539-2379163 | N/A |
| D7473 | 1 | *pneumophila* | Clinical | USA: Washington, Bellingham | 1977 | SAMN06928754 | CP021269 | 2383480-2386104 | [3] |
| **Allele 27** |  |  |  |  |  |  |  |  |  |
| ***D7475*** | 1 | *pneumophila* | Clinical | USA:Pennsylvania, Allentown | 1977 | SAMN06928755 | CP021283 | 2284367-2286991 | [3] |
| **Allele 28** |  |  |  |  |  |  |  |  |  |
| ***ST42*** | N/A | *N/A* | Clinical | [Germany](https://www.ncbi.nlm.nih.gov/biosample?term=) | 1999 | SAMEA4535099 | LT632617 | 2236544-2239168 | N/A |
| **Allele 29** |  |  |  |  |  |  |  |  |  |
| ***NCTC12179*** | 11 | *N/A* | Clinical | USA | 1982 | SAMEA3111979 | LR134176 | 1709454-1712078 | N/A |
| **Allele 30** |  |  |  |  |  |  |  |  |  |
| ***80-045*** | 1 | *N/A* | Clinical | Japan:Nagasaki | 1980 | N/A | AB107985 | 8927-11551 | [22] |
| **Allele 31** |  |  |  |  |  |  |  |  |  |
| ***ATCC43130*** | 11 | *pneumophila* | Clinical | USA: Newyork | 1982 | SAMN01917211 | HG531935 | 1-2625 | N/A |
| **Allele 32** |  |  |  |  |  |  |  |  |  |
| FFI337 | N/A | *pneumophila* | Environmental | Norway:Sarpsborg /Fredrikstad | 2008 | SAMN05513581 | CP016876 | 441391-444015 | [23] |
| FFI329 | N/A | *pneumophila* | Environmental | Norway:Sarpsborg /Fredrikstad | 2005 | SAMN05513580 | CP016874 | 2885788-2888409 | [23] |
| FFI105 | N/A | *pneumophila* | Environmental | Norway:Sarpsborg /Fredrikstad | 2008 | SAMN05513579 | CP016873 | 944336-946957 | [23] |
| FFI104 | N/A | *pneumophila* | Clinical | Norway | 2008 | SAMN05513578 | CP016872 | 3239073-3241694 | [23] |
| FFI102 | N/A | *pneumophila* | Clinical | Norway | 2005 | SAMN05513576 | CP016868 | 552329-554950 | [23] |
| ***Lens*** | 1 | *pneumophila* | Clinical | France:Lens | 2003 | SAMEA3138253 | CR628337 | 2351144-2353768 | [24] |
| FFI103 | N/A | *pneumophila* | Environmental | Norway:Sarpsborg /Fredrikstad | 2005 | SAMN05513577 | CP016870 | 1423915-1426538 | [23] |
| **Allele 33** |  |  |  |  |  |  |  |  |  |
| ***D5265*** | 1 | *raphaeli* | Clinical | USA:Pennsylvania, Philadelphia | 2002 | SAMN06928773 | CP021272 | 2424719-2427343 | [3] |
| D7787 | 5 | *raphaeli* | Clinical | USA: Texas, Houston | 2016 | SAMN06928774 | CP021270 | 2348563-2351187 | [3] |
| D4040 | 1 | *raphaeli* | N/A | USA: Delaware, Wilmington | 1994 | SAMN06928772 | CP021274 | 2424790-2427414 | [3] |
| **Allele 34** |  |  |  |  |  |  |  |  |  |
| ***D4954*** | N/A | *raphaeli* | Clinical | USA: Ohio, Hamilton | 2000 | SAMN06928771 | CP021256 | 2531686-2534310 | [3] |
| **Allele 35** |  |  |  |  |  |  |  |  | [3] |
| ***ATCC33737*** | 5 | *pascullei* | Environmental | USA | 1988 | SAMN01918939 | HG531950 | 1-2625 | [15] |
| **Allele 36** |  |  |  |  |  |  |  |  |  |
| ***ATCC33216*** | 5 | *fraseri* | Environmental | USA:Dallas | 1978 | SAMN05439986 | HG531953 | 1-2625 | [15] |
| **Allele 37** |  |  |  |  |  |  |  |  |  |
| ***F4198*** | 1 | *fraseri* | Environmental | USA:Pennsylvania, Pittsburgh | 2012 | SAMN06928761 | CP021279 | 2371913-2374537 | [3] |
| D4058 | 1 | *fraseri* | Clinical | USA: Connecticut, Bridgeport | 1994 | SAMN06928763 | CP021277 | 2447851-2450475 | [3] |
| Los Angeles 1 (D-7696) | 4 | *fraseri* | Clinical | USA: California, Los Angeles | 1978 | SAMN06928760 | CP021265 | 2430719-2433343 | [3] |
| D5387 | 1 | *fraseri* | Clinical | Denmark | 1998 | SAMN06928762 | CP021264 | 2382167-2384791 | [3] |
| D3137 | 1 | *fraseri* | Clinical | USA: California, Richmond | 1991 | SAMN06928764 | CP021263 | 2387096-2389720 | [3] |
| D5744 | 8 | *fraseri* | Clinical | USA: Arizona, Phoenix | 2008 | SAMN06928769 | CP021258 | 2397441-2400065 | [3] |
| D5945 | 1 | *fraseri* | Clinical | USA:New York, New York City | 2009 | SAMN05856000 | CP017602 | 2370751-2373375 | [3] |
| D6026 | 1 | *fraseri* | Clinical | USA: New York, New York City | 2016 | SAMN05856001 | CP017601 | 2369103-2371727 | [3] |
| Dallas 1E | 5 | *fraseri* | Environmental | USA:Dallas | 1978 | SAMN05818649 | CP017458 | 2402533-2405157 | [3] |
| Detroit-1 | 1 | *fraseri* | Clinical | USA:Michigan, Detroit | 1977 | SAMN05818658 | CP017457 | 2320537-2323161 | [25] |
| **Allele 38** |  |  |  |  |  |  |  |  |  |
| ***D7705*** | 1 | *raphaeli* | Clinical | USA: New York | 2007 | SAMN06928766 | CP021261 | 2439384-2442008 | [3] |
| D7706 | 1 | *raphaeli* | Environmental | [USA: New York](https://www.ncbi.nlm.nih.gov/biosample?term=) | 2007 | SAMN06928767 | CP021260 | 2474605-2477229 | [3] |
| **Allele 39** |  |  |  |  |  |  |  |  |  |
| ***D7708*** | 4 | *N/A* | Clinical | USA: Georgia, Atlanta | 2016 | SAMN06928768 | CP021259 | 2414771-2417395 | [3] |

*N/A indicates not available.

#Strain or sample names marked red indicate representative allele names.

**References**

1. Ginevra C, Forey F, Campese C, Reyrolle M, Che D, Etienne J, et al. Lorraine strain of Legionella pneumophila serogroup 1, France. Emerging infectious diseases. 2008;14(4):673-5. doi: 10.3201/eid1404.070961. PubMed PMID: 18394295; PubMed Central PMCID: PMC2570941.

2. Bissett ML, Lee JO, Lindquist DS. New serogroup of Legionella pneumophila, serogroup 8. Journal of clinical microbiology. 1983;17(5):887-91. Epub 1983/05/01. doi: 10.1128/JCM.17.5.887-891.1983. PubMed PMID: 6345579; PubMed Central PMCID: PMCPMC272760.

3. Kozak-Muiznieks NA, Morrison SS, Mercante JW, Ishaq MK, Johnson T, Caravas J, et al. Comparative genome analysis reveals a complex population structure of Legionella pneumophila subspecies. Infection, genetics and evolution : journal of molecular epidemiology and evolutionary genetics in infectious diseases. 2018;59:172-85. Epub 2018/02/11. doi: 10.1016/j.meegid.2018.02.008. PubMed PMID: 29427765.

4. Petzold M, Thurmer A, Menzel S, Mouton JW, Heuner K, Luck C. A structural comparison of lipopolysaccharide biosynthesis loci of Legionella pneumophila serogroup 1 strains. BMC microbiology. 2013;13:198. doi: 10.1186/1471-2180-13-198. PubMed PMID: 24069939; PubMed Central PMCID: PMC3766260.

5. Rao C, Guyard C, Pelaz C, Wasserscheid J, Bondy-Denomy J, Dewar K, et al. Active and adaptive Legionella CRISPR-Cas reveals a recurrent challenge to the pathogen. Cellular microbiology. 2016;18(10):1319-38. Epub 2016/03/05. doi: 10.1111/cmi.12586. PubMed PMID: 26936325; PubMed Central PMCID: PMCPMC5071653.

6. Borges V, Nunes A, Sampaio DA, Vieira L, Machado J, Simoes MJ, et al. Legionella pneumophila strain associated with the first evidence of person-to-person transmission of Legionnaires' disease: a unique mosaic genetic backbone. Scientific reports. 2016;6:26261. Epub 2016/05/20. doi: 10.1038/srep26261. PubMed PMID: 27196677; PubMed Central PMCID: PMCPMC4872527.

7. Hookey JV, Birtles RJ, Saunders NA. Intergenic 16S rRNA gene (rDNA)-23S rDNA sequence length polymorphisms in members of the family Legionellaceae. Journal of clinical microbiology. 1995;33(9):2377-81. Epub 1995/09/01. doi: 10.1128/JCM.33.9.2377-2381.1995. PubMed PMID: 7494031; PubMed Central PMCID: PMCPMC228416.

8. Kozak-Muiznieks NA, Lucas CE, Brown E, Pondo T, Taylor TH, Jr., Frace M, et al. Prevalence of sequence types among clinical and environmental isolates of Legionella pneumophila serogroup 1 in the United States from 1982 to 2012. Journal of clinical microbiology. 2014;52(1):201-11. doi: 10.1128/JCM.01973-13. PubMed PMID: 24197883; PubMed Central PMCID: PMC3911437.

9. Mercante JW, Morrison SS, Desai HP, Raphael BH, Winchell JM. Genomic Analysis Reveals Novel Diversity among the 1976 Philadelphia Legionnaires' Disease Outbreak Isolates and Additional ST36 Strains. PloS one. 2016;11(9):e0164074. Epub 2016/09/30. doi: 10.1371/journal.pone.0164074. PubMed PMID: 27684472; PubMed Central PMCID: PMCPMC5042515.

10. Buultjens AH, Chua KYL, Baines SL, Kwong J, Gao W, Cutcher Z, et al. A Supervised Statistical Learning Approach for Accurate Legionella pneumophila Source Attribution during Outbreaks. Applied and environmental microbiology. 2017;83(21). Epub 2017/08/20. doi: 10.1128/AEM.01482-17. PubMed PMID: 28821546; PubMed Central PMCID: PMCPMC5648911.

11. Khan MA, Knox N, Prashar A, Alexander D, Abdel-Nour M, Duncan C, et al. Comparative Genomics Reveal That Host-Innate Immune Responses Influence the Clinical Prevalence of Serogroups. PloS one. 2013;8(6):e67298. doi: 10.1371/journal.pone.0067298. PubMed PMID: 23826259; PubMed Central PMCID: PMC3694923.

12. Ma J, He Y, Hu B, Luo ZQ. Genome Sequence of an Environmental Isolate of the Bacterial Pathogen Legionella pneumophila. Genome announcements. 2013;1(3). Epub 2013/06/26. doi: 10.1128/genomeA.00320-13. PubMed PMID: 23792742; PubMed Central PMCID: PMCPMC3675512.

13. Amaro F, Gilbert JA, Owens S, Trimble W, Shuman HA. Whole-genome sequence of the human pathogen Legionella pneumophila serogroup 12 strain 570-CO-H. J Bacteriol. 2012;194(6):1613-4. doi: 10.1128/JB.06626-11. PubMed PMID: 22374950; PubMed Central PMCID: PMC3294838.

14. Chien M, Morozova I, Shi S, Sheng H, Chen J, Gomez SM, et al. The genomic sequence of the accidental pathogen Legionella pneumophila. Science. 2004;305(5692):1966-8. Epub 2004/09/28. doi: 10.1126/science.1099776. PubMed PMID: 15448271.

15. Costa J, Teixeira PG, d'Avo AF, Junior CS, Verissimo A. Intragenic recombination has a critical role on the evolution of Legionella pneumophila virulence-related effector sidJ. PloS one. 2014;9(10):e109840. doi: 10.1371/journal.pone.0109840. PubMed PMID: 25299187; PubMed Central PMCID: PMCPMC4192588.

16. Gomez-Valero L, Rusniok C, Jarraud S, Vacherie B, Rouy Z, Barbe V, et al. Extensive recombination events and horizontal gene transfer shaped the Legionella pneumophila genomes. BMC genomics. 2011;12:536. doi: 10.1186/1471-2164-12-536. PubMed PMID: 22044686; PubMed Central PMCID: PMC3218107.

17. Merrell DS, Sahl J, Shames SR, Costa J, Sousa PS, Silva IN, et al. Differences in Virulence BetweenLegionella pneumophilaIsolates From Human and Non-human Sources Determined inGalleria mellonellaInfection Model. 2018;8:97-.

18. Ginevra C, Jacotin N, Diancourt L, Guigon G, Arquilliere R, Meugnier H, et al. Legionella pneumophila sequence type 1/Paris pulsotype subtyping by spoligotyping. Journal of clinical microbiology. 2012;50(3):696-701. doi: 10.1128/JCM.06180-11. PubMed PMID: 22205819; PubMed Central PMCID: PMC3295150.

19. D'Auria G, Jimenez-Hernandez N, Peris-Bondia F, Moya A, Latorre A. Legionella pneumophila pangenome reveals strain-specific virulence factors. BMC genomics. 2010;11:181. doi: 10.1186/1471-2164-11-181. PubMed PMID: 20236513; PubMed Central PMCID: PMC2859405.

20. Schroeder GN, Petty NK, Mousnier A, Harding CR, Vogrin AJ, Wee B, et al. Legionella pneumophila strain 130b possesses a unique combination of type IV secretion systems and novel Dot/Icm secretion system effector proteins. J Bacteriol. 2010;192(22):6001-16. doi: 10.1128/JB.00778-10. PubMed PMID: 20833813; PubMed Central PMCID: PMC2976443.

21. Kozak-Muiznieks NA, Morrison SS, Sammons S, Rowe LA, Sheth M, Frace M, et al. Three Genome Sequences of Legionella pneumophila subsp. pascullei Associated with Colonization of a Health Care Facility. Genome announcements. 2016;4(3). Epub 2016/05/07. doi: 10.1128/genomeA.00335-16. PubMed PMID: 27151801; PubMed Central PMCID: PMCPMC4859183.

22. Chang B, Kura F, Amemura-Maekawa J, Koizumi N, Watanabe H. Identification of a novel adhesion molecule involved in the virulence of Legionella pneumophila. Infection and immunity. 2005;73(7):4272-80. Epub 2005/06/24. doi: 10.1128/IAI.73.7.4272-4280.2005. PubMed PMID: 15972519; PubMed Central PMCID: PMCPMC1168565.

23. Dybwad M, Aarskaug T, Fykse EM, Henie Madslien E, Blatny JM. Complete Genome Sequences of Six Legionella pneumophila Isolates from Two Collocated Outbreaks of Legionnaires' Disease in 2005 and 2008 in Sarpsborg/Fredrikstad, Norway. Genome announcements. 2016;4(6). Epub 2016/12/17. doi: 10.1128/genomeA.01367-16. PubMed PMID: 27979936; PubMed Central PMCID: PMCPMC5159569.

24. Newton HJ, Ang DK, van Driel IR, Hartland EL. Molecular pathogenesis of infections caused by Legionella pneumophila. Clinical microbiology reviews. 2010;23(2):274-98. Epub 2010/04/09. doi: 10.1128/CMR.00052-09. PubMed PMID: 20375353; PubMed Central PMCID: PMCPMC2863363.

25. Raphael BH, Kozak-Muiznieks NA, Morrison SS, Mercante JW, Winchell JM. Complete Genome Sequences of Legionella pneumophila subsp. fraseri Strains Detroit-1 and Dallas 1E. Genome announcements. 2017;5(5). Epub 2017/02/06. doi: 10.1128/genomeA.01525-16. PubMed PMID: 28153889; PubMed Central PMCID: PMCPMC5289675.
