# Supplementary material for: Virulence effector SidJ evolution in Legionella pneumophila is driven by positive selection and intragenic recombination
Source: PeerJ. 2021 Aug 17;9:e12000. doi: 10.7717/peerj.12000 (PMC8378335; doi:10.7717/peerj.12000)
Supplement: Supplemental Information 2 — P is the number of parameters in the ω distribution; lnL is the log likelihood; ω is ratio of dN/dS, LRT P-value indicates the value of chi-square test; Parameters indicating positive selection are presented in bold; positive selection sites were identified by the Bayes empirical Bayes (BEB) methods under M8 model or by Naive Empirical Bayes (NEB) methods under M3 and M2a models. The posterior probabilities (p) ≥ 0.90, (p) ≥ 0.95 and p ≥ 0.99 are indicated by *, ** and ***, respectively. Yang et al. recommended that results from M8 model were preferred to find sites under positive selection pressure, and it is more robust to recombination which was proved by Maria et al. [file peerj-09-12000-s002.docx]

**Table S2. Log-likelihood values and parameter estimates for the *sidJ* gene of *L. pneumophila* using unmodified topology tree of the alleles.**

| Model | *nP* | *lnL* | Estimates of parameters | LRT P-value | Positively sites |
| --- | --- | --- | --- | --- | --- |
| M3 (discrete) | 81 | -8714.6954 | p0=0.7795, p1=0.2133, p**2=0.00723**, ω0=0.01729,  ω1=0.7069, **ω2=3.7116** | P <10^-9^ | **58G*****, 200N*, 868T*, 869S* |
| M0 (one ratio) | 77 | -8863.1637 | ω0=0.1732 |  | Not Allowed |
| M2a (selection) | 80 | -8717.1477 | p0=0.8370, p1=0.1587, **p2=0.00431**, ω0=0.03499, ω1=1.00000, **ω2=4.5150** | P =0.011129923 | **N/A** |
| M1a (neutral) | 78 | -8721.6458 | p0=0.8375, p1=0.1625  ω0=0.03404, ω1=1.0000 |  | Not Allowed |
| M8^a^ (beta&ω) | 80 | -8718.3447 | p0=0.9844, p=0.03938, q=0.1953  **p1=0.0156, ω=2.7132** | P =0.000000637 | **58G**, 200N**,** 868T***, 869S**** |
| M7 (beta) | 78 | -8732.6114 | p=0.03809, q=0.17020 |  | Not Allowed |

*P* is the number of parameters in the ω distribution; lnL is the log likelihood; ω is ratio of *dN*/*dS*, LRT P-value indicates the value of chi-square test; Parameters indicating positive selection are presented in bold; Positive selection sites were identified by the Bayes empirical Bayes (BEB) methods under M8 model or by Naive Empirical Bayes (NEB) methods under M3 and M2a models. The posterior probabilities(p)≥0.90, (p)≥0.95 and p≥0.99 are indicated by *, ** and ***, respectively. Yang *et al.* recommended that results from M8 model were preferred to find sites under positive selection pressure, and it is more robust to recombination which was proved by Maria et al.
