# Supplementary material for: Virulence effector SidJ evolution in Legionella pneumophila is driven by positive selection and intragenic recombination
Source: PeerJ. 2021 Aug 17;9:e12000. doi: 10.7717/peerj.12000 (PMC8378335; doi:10.7717/peerj.12000)
Supplement: Supplemental Information 3 — α indicates(dS) value and β or β+ indicates(dN) values. # Cut-off value for P-values in MEMER was set at 0.03 to enhance the analysis specificity . *Cut-off value for Posterior Pr in FUBAR was set at 0.90. [file peerj-09-12000-s003.docx]

**Table S3.Parameter estimates for the *sidJ* gene of *L.pneumophil*a and positive selection sites detected by methods implemented in HyPhy package.**

| **Methods& algorithms** |  |  |  |  |
| --- | --- | --- | --- | --- |
| ***MEME** |  |  |  |  |
| **Positive selection sites** | **α** | **β+** | **LRT** | **P-value** |
| 37 | 0.00 | 62.37 | 15.41 | <0.01 |
| ***58*** | ***0.00*** | ***3.87*** | ***6.10*** | ***0.02*** |
| 119 | 0.00 | 325.68 | 5.49 | 0.03 |
| 250 | 0.00 | 529.69 | 7.99 | <0.01 |
| 256 | 0.00 | 107.19 | 11.73 | <0.01 |
| 258 | 1.14 | 932.00 | 10.93 | <0.01 |
| 259 | 0.00 | 110.89 | 8.43 | <0.01 |
| 262 | 0.00 | 666.43 | 16.12 | <0.01 |
| 648 | 0.00 | 221.62 | 6.24 | 0.02 |
| 725 | 0.00 | 69.96 | 9.23 | 0.00 |
| 867 | 1.56 | 47.85 | 7.94 | 0.01 |
| ***868*** | ***5.22*** | ***236.33*** | ***5.21*** | ***0.03*** |
| ***869*** | ***0.00*** | ***31.48*** | ***6.93*** | ***0.01*** |
| **FEL** |  |  |  |  |
| **Positive selection sites** | **α** | **β** | **ω** | **P-value** |
| ***58*** | ***0.000*** | ***2.527*** | ***Infinity*** | ***0.043*** |
| 820 | 0.000 | 2.497 | Infinity | 0.034 |
| ***869*** | ***0.000*** | ***3.233*** | ***Infinity*** | ***0.015*** |
| **FUBAR** |  |  |  |  |
| **Positive selection sites** | **α** | **β** | **Bayes Factor[β>α]** | **Posterior Pr[β>α]** |
| ***58*** | ***0.644*** | ***5.349*** | ***71.913*** | ***0.965*** |
| ***869*** | ***0.756*** | ***2.848*** | ***24.574*** | ***0.904#*** |
| **Evolutionary Fingerprinting** |  |  |  |  |
| **Positive selection sites** | **α** | **β** | **Bayes Factor[β>α]** | **Posterior Pr[β>α]** |
| ***58*** | ***0.334*** | ***2.552*** | ***460.531*** | ***0.9976*** |
| ***200*** | ***0.426*** | ***2.493*** | ***27.270*** | ***0.9610*** |
| 820 | 0.417 | 2.161 | 28.783 | 0.9630 |
| ***869*** | ***0.340*** | ***2.550*** | ***249.531*** | ***0.9956*** |

α indicates (dS) value and  β or β+ indicates (dN) values.

# Cut-off value for P-values in MEMER was set at 0.03 to enhance the analysis specificity .

*Cut-off value for Posterior Pr in FUBAR was set at 0.90.
