# Supplementary material for: Virulence effector SidJ evolution in Legionella pneumophila is driven by positive selection and intragenic recombination
Source: PeerJ. 2021 Aug 17;9:e12000. doi: 10.7717/peerj.12000 (PMC8378335; doi:10.7717/peerj.12000)
Supplement: Supplemental Information 4 — PNA: P olar n eutral a mino acidPAA: P olar a cidic a mino acidPBA: P olar b asic a mino acidNPA: Non-p olar a mino acid*Underlines indicate that these mutation profiles have corresponding amino acid substitution model. [file peerj-09-12000-s004.docx]

**Table S4. Mutation profiles of positive selection sites in SidJ.**

| Positive selection sites | Mutation profiles | Amino acid substitution model | Percentage of the alleles with mutation profiles (%) |
| --- | --- | --- | --- |
| 58 | G58R, G58M,G58E* | NPA to PBA, to PNA and to PAA | 43.59 |
| 200 | N200T, N200I, N200A and N200V | PNA to NPA | 71.79 |
| 868 | T868N and T686P | PNA to NPA | 28.20 |
| 869 | S869T and S869P | PNA to NPA | 35.90 |

PNA: *P*olar *n*eutral *a*mino acid

PAA: *P*olar *a*cidic *a*mino acid

PBA: *P*olar *b*asic *a*mino acid

NPA: Non-*p*olar *a*mino acid

*Underlines indicate that these mutation profiles have corresponding amino acid substitution model.
