# Supplementary material for: Virulence effector SidJ evolution in Legionella pneumophila is driven by positive selection and intragenic recombination
Source: PeerJ. 2021 Aug 17;9:e12000. doi: 10.7717/peerj.12000 (PMC8378335; doi:10.7717/peerj.12000)
Supplement: Supplemental Information 5 — * Fisher’s exact test was used for comparing distribution differences of mutation profiles in different types of sidJ alleles. # The red color indicates mutation profiles have distinct distribution between different types of sidJ alleles. [file peerj-09-12000-s005.docx]

**Table S5.Positive selection sites codon distribution patterns in different types of alleles.**

| Codon | **58** | | | | **200** | | | | | **868** | | | **869** | | |
| --- | --- | --- | --- | --- | --- | --- | --- | --- | --- | --- | --- | --- | --- | --- | --- |
| Mutation Profiles | 58G | G58R | G58M# | G58E | T200N | 200T | T200I | T200A | T200V | 868T | T868N | T868P | 869T | T869S | T869P |
| CA, n (%) | 17 (62.96%) | 4 (14.81%) | 5 (18.52%) | 1 (3.70%) | 6 (22.22%) | 7 (25.92%) | 2 (7.41%) | 11 (40.74%) | 1 (3.70%) | 18 (66.67%) | 3 (11.11%) | 6 (22.22%) | 5 (18.52%) | 16 (59.26%) | 6 (22.22%) |
| EA, n (%) | 5 (41.67%) | 0 (0%) | 7 (58.33%) | 0 (0%) | 3 (25%) | 5 (41.67%) | 0 (0%) | 4 (33.33%) | 0 (0%) | 10 (83.33%) | 1 (8.33%) | 1 (8.33%) | 2 (16.67%) | 9 (75%) | 1 (8.33%) |
| *P-Values* | 0.216 | 0.213* | 0.013 | 0.692 | 0.576* | 0.326 | 0.474* | 0.472* | 0.692* | 0.253* | 0.640* | 0.289* | 0.635* | 0.283 | 0.289* |
| Recombinant, n (%) | 8 (57.14%) | 2 (14.29%) | 3 (21.43%) | 1 (7.14%) | 0 (0%) | 5 (35.71%) | 0 (%) | 8 (57.14%) | 1 (7.14) | 8 (57.14%) | 1 (7.14%) | 5 (35.71%) | 0 (0%) | 9 (64.29%) | 5 (35.71%) |
| Non-recombinant, n (%) | 14 (56%) | 2 (8%) | 9 (36%) | 0 (0%) | 9 (36%) | 7 (28%) | 2 (8%) | 7 (28%) | 0 (0) | 20 (80%) | 3 (12%) | 2 (8%) | 7 (28%) | 16 (64%) | 2 (8%) |
| *P-Values* | 0.945 | 0.455* | 0.283* | 0.359* | 0.010* | 0.440 | 0.405 | 0.073 | 0.359* | 0.128 | 0.545* | 0.044* | 0.031* | 0.986 | 0.044* |

CA: clinically associated alleles

EA: environmental alleles

* Fisher’s exact test was used for comparing distribution differences of mutation profiles in different types of *sidJ* alleles.

# The red color indicates mutation profiles have distinct distribution between different types of *sidJ* alleles.
