# Supplementary material for: Virulence effector SidJ evolution in Legionella pneumophila is driven by positive selection and intragenic recombination
Source: PeerJ. 2021 Aug 17;9:e12000. doi: 10.7717/peerj.12000 (PMC8378335; doi:10.7717/peerj.12000)
Supplement: Supplemental Information 6 [file peerj-09-12000-s006.docx]

**Table S6. Information of CaM protein in different species of potential *L. pneumophila* hosts.**

| Accession No. | Protein name | Species / strain names | Protein length (aa) * |
| --- | --- | --- | --- |
| AAD45181.1 | CaM | *Homo sapiens* | 149 |
| XP_001022775.2 | CaM | *Tetrahymena thermophila SB210* | 149 |
| AAA33172.1 | CaM | *Dictyostelium discoideum* | 152 |
| ELR14060.1 | CaM putative 1 | *Acanthamoeba castellanii str. Neff* | 149 |
| XP_004336073.1 | CaM putative 1 | *Acanthamoeba castellanii str. Neff* | 149 |
| KYQ99709.1 | CaM | *Tieghemostelium lacteum* | 151 |
| XP_003293234.1 | CaM | *Dictyostelium purpureum* | 151 |
| XP_004361968.1 | CaM | *Cavenderia fasciculata* | 143 |
| XP_002674748.1 | CaM | *Naegleria gruberi* | 144 |
| XP_009308421.1 | CaM | *Trypanosoma_grayi* | 145 |
| XP_001740593.1 | CaM putative 1 | *Entamoeba dispar SAW760* | 146 |
| XP_652365.1 | CaM putative 1 | *Entamoeba histolytica HM-1:IMSS* | 146 |
| GAT99218.1 | CaM putative | *Entamoeba histolytica* | 150 |
| XP_001739364.1 | CaM putative 2 | *Entamoeba dispar SAW760* | 150 |
| XP_655757.1 | CaM putative 2 | *Entamoeba histolytica HM-1:IMSS* | 150 |
| XP_001734679.1 | CaM putative 3 | *Entamoeba dispar SAW760* | 151 |
| XP_651708.1 | CaM putative 3 | *Entamoeba histolytica HM-1:IMSS* | 151 |
| XP_001737671.1 | CaM putative 4 | *Entamoeba dispar SAW760* | 144 |
| XP_652048.1 | CaM putative 4 | *Entamoeba histolytica HM-1:IMSS* | 144 |
| XP_004334690.1 | CaM putative 3 | *Acanthamoeba castellanii str. Neff* | 154 |
| XP_004337386.1 | CaM putative 4 | *Acanthamoeba castellanii str. Neff* | 153 |

* indicates number of amino acid.
